# Influence of Amiodarone and Dronedarone on the Force-Interval Dependence of Rat Myocardium

**DOI:** 10.1155/2018/4737489

**Published:** 2018-08-02

**Authors:** Dina S. Kondratieva, Sergey A. Afanasiev, Sergey V. Popov

**Affiliations:** Cardiology Research Institute, Tomsk NRMC, Russia

## Abstract

The antiarrhythmic effect of amiodarone and its analogue dronedarone is caused by their direct actions on several cardiomyocyte sarcolemmal ion currents. However, whether their effects are related to intracellular calcium levels is not exactly known. Ca^2+^ cycling refers to the release and reuptake of intracellular Ca^2+^, which induces muscle contraction and relaxation and determines the force-interval dependence. This study aimed to evaluate the influence of amiodarone and dronedarone on the force-interval relationship.* Materials and Results*. The work was performed on the papillary muscles of the left ventricle of male Wistar rats. Muscle perfusion was performed at 36.5°C with oxygenated Krebs-Henseleit solution with baseline stimulation 0.5 Hz. The postrest test (4-60 s) and the extrasystolic exposure (0.2-1.5 s) were evaluated. Inotropic reaction to the test exposure was evaluated before and after muscle perfusion with solution containing amiodarone (10^−6^ M) or dronedarone (10^−6^ M) during 10 min. Amiodarone or dronedarone led to decrease of the amplitude of extrasystolic contractions of the papillary muscles. The amplitude of postextrasystolic contractions after short extrasystolic intervals on the background of the drugs was increased. Amiodarone and dronedarone led to increase of the amplitude of postrest contractions.* Conclusions*. Dronedarone reduces the excitability of cardiomyocyte sarcolemma to a greater extent than amiodarone. Amiodarone and dronedarone are able to increase postextrasystolic and postrest potentiation. The effect of amiodarone on postextrasystolic and postrest potentiation is more pronounced in comparison with dronedarone.

## 1. Introduction

At the present time amiodarone is the most effective antiarrhythmic drug for the treatment of ventricular and supraventricular arrhythmias [1–3]. It is a class III antiarrhythmic drug whose main effect occurs through the blockade of potassium channels [4]. In addition, this drug possesses antiadrenergic activity and can block fast sodium and slow calcium channels [5, 6]. However, application of amiodarone is limited by the possible development of complications [7–9]. In this connection development of new drugs that are similar to amiodarone but devoid of its side effects comes at an opportune time. Recently, new preparation of dronedarone whose chemical structure is similar to amiodarone has been proposed. This drug does not contain the iodinated residues that decreases thyroid effects that are inherent to amiodarone [10, 11]. Despite the similarity in structure and electrophysiological properties of both drugs, amiodarone is more efficient antiarrhythmic agent [12]. Recently, we showed that amiodarone is able to increase postrest contraction amplitude of isolated myocardium strips [13]. As it is known potentiation effect of postrest contraction is connected to functional activity of the sarcoplasmic reticulum (SR) [14]. It is probable that high efficiency of amiodarone may be caused by its influence on the Ca^2+^ transport systems of the SR. The functional consistency of the Ca^2+^ transport systems of the SR is important component in the maintenance of intracellular Ca^2+^ homeostasis [15, 16]. So, disorders of Ca^2+-^ATPase activity of the SR deteriorate relaxation of cardiomyocyte [17–20] and pathological changes in the function of calcium channels of the SR (ryanodine receptors) lead to increase of spontaneous diastolic release of calcium ions [21, 22]. Spontaneous release of calcium ions increases transient ion currents which leads to appearance of delayed postdepolarization and following triggered activity of cardiomyocytes [23–25]. It is known that amiodarone and dronedarone prevent appearance of such arrhythmias. However the present ability of these drugs to influence intracellular transport of calcium ions by the SR is studied insufficiently.

In this work, the influence of amiodarone and dronedarone on the of force-interval dependence dynamics of the rat myocardium was studied.

## 2. Methods and Materials

### 2.1. Muscle Preparation

All procedures with experimental animals were performed in accordance with “Rules of Working with and Use of Experimental Animals” from the Order of the Ministry of Health of the USSR No. 755 from August 12, 1977. The study was performed using 22 intact male Wistar rats (body weight 180–200 g). Animals were immobilized by shifting of the cervical spine under light ether anaesthesia. The thorax was lanced and the heart was separated and placed into ice-cold Krebs-Henseleit solution (the composition is provided below). The papillary muscles, with cross-sectional diameter of 0.5 ± 0.7 mm, were separated from the left ventricle. The prepared muscles were placed in a 1 ml thermos stabilized (36 ± 0.5°C) flow chamber to study the contractile activity of isolated muscle preparations (Scientific Instruments GmbH, Germany). Muscles perfusion was performed with Krebs-Henseleit solution of the following composition (in mM): NaCl 120; KCl 4.8; CaCl_2_ 2.0; MgSO_4_ 1.2; KH_2_PO_4_ 1.2; NaHCO_3_ 20.0; glucose 10.0. Oxygenation of solution was performed with carbogen (O_2_-95%, CO_2_-5%).

### 2.2. Tension Measurement

The contractile activity of the muscles was estimated in isometric mode, using force transducer KG-Series sensor (Scientific Instruments GmbH). Basic stimulation of the muscles was performed with 5 ms rectangular electrical pulses at frequency of 0.5 Hz. The muscles were stretched to the length at which the contraction was maximal (Lmax). All muscles were adapted to perfusion conditions and isometric mode during 60 min. Isometric tension was evaluated by force normalized to the muscle cross-sectional area (mN/mm^2^) and the following parameters were obtained: force twitches amplitude (Tmax), the velocity of developed tension (+ dT/dt), the velocity of relaxation (-dT/dt), contraction-relaxation cycle period (t), time of developed tension (t1), and time of relaxation (t2).

### 2.3. Extrasystolic Test

**Figure 1:**
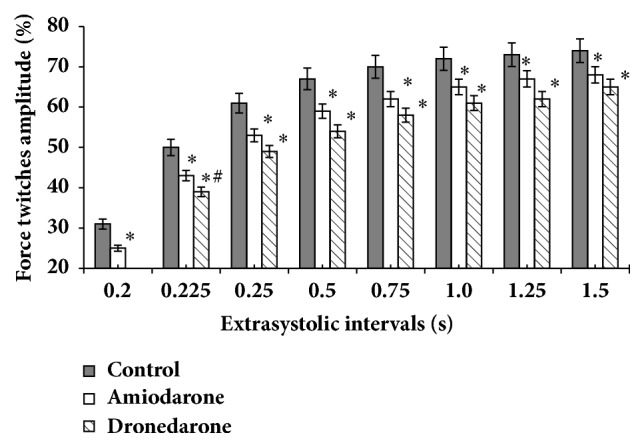
Effects of amiodarone and dronedarone on the extrasystolic contractions. Note. X-axis: extrasystolic intervals (s); Y-axis: force twitches amplitude expressed as a percentage to amplitude of baseline contractions. *∗*  p < 0.05 versus baseline contractions (control). #  p < 0.05 versus amiodarone.

Extraordinary electrical rectangular pulses with 5 ms width were delivered to induce extrasystolic contraction. When the muscle contractions had reached steady state, extrasystolic contraction was caused by a single application of the extraordinary electrical pulse in 0.2, 0.225, 0.25, 0.5, 0.75, 1.0, 1.25, and 1.5 s after the start of the steady state cycle. The amplitudes of extrasystolic and postextrasystolic inotropic response were estimated as percentage of the values of the regular contraction-relaxation cycle (Figure 1). Changing of extrasystolic inotropic response to extraordinary electrical pulse characterizes sarcolemma excitability [26]. The postextrasystolic contraction amplitude indirect characterizes ability of the SR to accumulate additional amount Са^2+^ ions influxed into the myoplasm during extraordinary excitation [27].

### 2.4. Postrest Test

The postrest test was performed on the background of the basic stimulation frequency. Electrical stimulation of the muscles was switched off for 4, 6, 8, 10, 12, 16, 20, 30, and 60 s (rest periods). The amplitude of the regular cycle and the first contraction-relaxation cycle after each rest period was measured. The dynamics of mechanical restitution was estimated as the dependence of the amplitude of the first contraction-relaxation cycle after rest period on the duration of these periods [14]. It has been shown that such methodical approach allows the estimation of the ability of the cardiomyocyte SR to release and to reuptake Ca^2+^ during a single contraction-relaxation cycle.

To estimate recycling fraction of calcium ions we performed calculation of fall coefficient of inotropic response potentiated with rest period at 60 s [28, 29]. For that we built linear regression of the amplitude of the n-th contraction cycle versus amplitude of the n+1-th contraction [28, 29]. Angle of slope of the obtained diagram was fall coefficient.

### 2.5. Experimental Protocol

Control recording of the contractile activity of the muscle and their initial reactions to extrasystolic and postrest exposure were performed after their adaptation to the stimulation mode during 60 minutes. After restoring the muscle contractile activity from test exposure, the muscle perfusion with solution containing amiodarone or dronedarone was performed during 10 minutes. After stabilizing effect of the drugs on the contractile activity of the muscles, the test exposure was repeated and their inotropic reaction was recorded.

### 2.6. Chemicals

Amiodarone (2-{4-[(2-butyl-1-benzofuran-3-yl)carbonyl]-2,6-diiodophenoxy}ethyl) diethylamine) (Sanofi-Aventis, France) was used in the dose of 10^−6^ M [30Ugdyzhekova].

Dronedarone (Dronedaronum)-N-(2-Butyl-3-(p-[3-(dibutylamino)propoxy]benzoyl)-5-benzofuranyl)methanesulfonamide (Multaq, Sanofi-Aventis, France) was used in the dose of 10^−6^ M.

### 2.7. Statistical Analysis

The statistical significance of drug effects was estimated by the nonparametric Wilcoxon test and reliability of distinctions between groups was estimated by the nonparametric Mann-Whitney U criterion. Differences at the P-value < 0.05 were considered significant. Data are expressed as mean ± standard error of the mean (SEM).

## 3. Results

### 3.1. Influence of Amiodarone and Dronedarone on the Parameters of the Single Cycle of Contraction-Relaxation

Table 1 presents the changes in the parameters of the single contraction-relaxation cycle of the rat papillary muscle after exposure to the drugs that were used. The perfusion of papillary muscles with solution containing amiodarone led to statistically significant changes in a number of parameters. Thus, the force twitches amplitude (Tmax) decreased by 10% and 8%, the velocities of developed tension (+dT/dt), and relaxation (-dT/dt) decreased by 8 and 13%, respectively. At the same time, such parameters as contraction-relaxation cycle period (t), time of developed tension (t1), and time of relaxation (t2) were not changed significantly.

Perfusion of the papillary muscles with a solution containing dronedarone also caused changes in the parameters of single contraction-relaxation cycle. At the same time, as with use of amiodarone, values of such indicators as Tmax, + dT/dt, and -dT/dt had statistically significant decrease by 8%, 11 %, and 19 %, respectively. In addition, the contraction-relaxation cycle period (t) and the time of relaxation (t2) were increased by 14 % and 16%, respectively. The time of developed tension (t1) remained unchanged.

### 3.2. Effect of Amiodarone and Dronedarone on Extrasystolic Contraction

The results obtained during extrasystolic test show that intact papillary muscles exhibit a typical reaction on extraordinary (extrasystolic) electrical stimulus (Figure 1). At the extrasystolic intervals 0.225, 0.25, and 0.5 s the extrasystolic contraction increased statistically significantly. However, a further increase in the extrasystolic interval duration did not lead to significant increase in the extrasystolic contraction amplitude (Figure 1).

On the background of amiodarone, extraordinary stimulation of the muscles caused extrasystolic inotropic response of lower amplitude than in the control conditions. Decrease of the amplitude of the extrasystolic inotropic response over the entire range of extrasystolic intervals was 6-8% (Figure 1), which was statistically significant (p < 0.05).

Perfusion of the muscles with dronedarone also promoted decrease of extrasystolic inotropic response amplitude compared to similar stimuli under control conditions (Figure 1). At that, effect of decrease of amplitudes was observed at all EIs and this difference was statistically significant. Moreover, we revealed that extrasystolic intervals with length of 0.2–0.5 s were expressed statistically significantly (p < 0.05) more than on the amiodarone background.

### 3.3. The Effect of Amiodarone and Dronedarone on Postextrasystolic Potentiation

**Figure 2:**
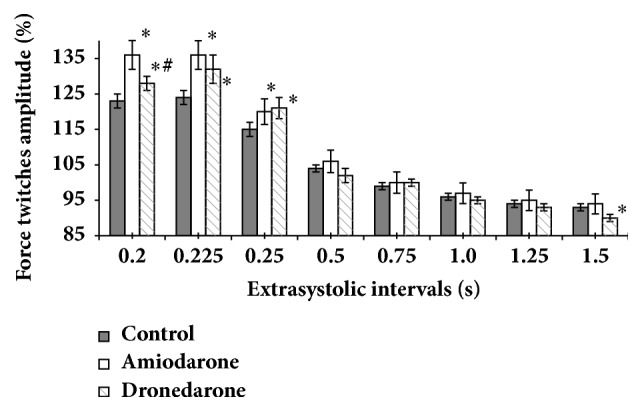
Effects of amiodarone and dronedarone on the force twitches amplitude of the postextrasystolic contractions. *∗*  p < 0.05 versus control. # p < 0.05 versus amiodarone.

Figure 2 presents data reflecting the magnitude of postextrasystolic inotropic response of papillary muscles in dependence on extrasystolic interval duration. It can be seen that, in the intact state at extrasystolic intervals of 0.2, 0.225, and 0.25 s, postextrasystolic contraction was statistically significantly higher than baseline contractions (p < 0.05); i.e., the effect of potentiation was observed. Expressiveness of this effect was decreased with elongation of EI. At extrasystolic interval with duration of 0.5 s, the potentiation effect was absent. With increase of the interval, inotropic response to the extraordinary stimuli became not much less than baseline contractions. Such a result coincides with the already described regularities [27].

After treatment the muscles with amiodarone, potentiation of the inotropic response was statistically significantly higher than indices obtained for intact muscles (Figure 2). After short EI (0.2 and 0.225 s), postextrasystolic inotropic response on the background of amiodarone was statistically significantly (p < 0.05) higher than the amplitude of regular inotropic response by 36 and 24%, respectively. At that, on the background of amiodarone, excess of the values of postextrasystolic potentiation of intact muscles was 13 and 12% (p < 0.05). After longer extrasystolic intervals expressiveness of postextrasystolic inotropic reactions has practically coincided with one of intact muscles.

The treatment of dronedarone also contributed to a reliable increase of amplitude of postextrasystolic contractions after short EI (Figure 2). Thus, at extrasystolic intervals of 0.2, 0.225, and 0.25 s, the amplitude of postextrasystolic contraction exceeded similar indices of intact muscles by 5, 8, and 6% (p < 0.05), respectively. At that, the potentiating effect of the muscles treated with dronedarone in these extrasystolic intervals was less than one with use of amiodarone. After long extrasystolic intervals we did not received significant differences in the inotropic reaction of intact muscles and muscles treated with dronedarone, except one for the longest EI of 60 s.

### 3.4. The Effect of Amiodarone and Dronedarone on Postrest Contractions

**Figure 3:**
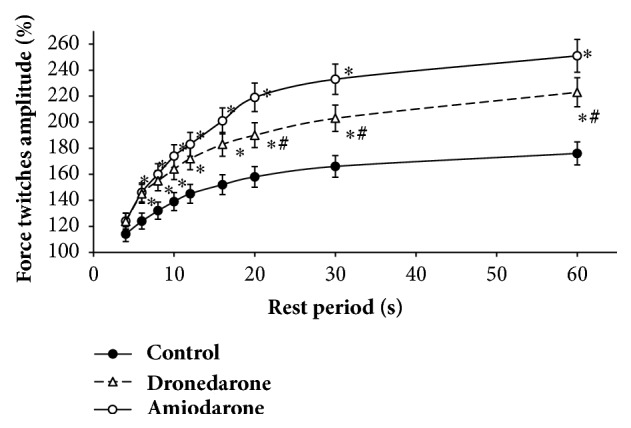
Mechanical restitution of the papillary muscles in the presence of amiodarone and dronedarone. *∗*p < 0.05 versus control. # p < 0.05 versus amiodarone.

As we can see in Figure 3, dependence of contraction amplitude of intact muscles on rest periods had typical positive trend. So, after rest period with duration of 4 s, increase of inotropic response was 24% (p < 0.05). For periods of 20 s, the increase was already over 80% (p < 0.05). However, subsequent increase of rest period duration up to 30 s and further up to 60 s did not lead to significant increase of inotropic response. This dependence is consistent with the previously described reactions of rat myocardium for execution of postrest test [14].

Muscles treated with amiodarone retained and even enhanced effect of inotropic response potentiation with increase of the duration of rest periods and over entire range of exposures. So, on the background amiodarone with the rest period of 4 s induced inotropic response that exceeded one of intact muscles by 10%. With rest period of 6 s, difference with inotropic reaction of intact muscles increased up to 28% (p < 0.05). After a rest period of 20 s, the difference in the inotropic response was already over 60% (p < 0.05). However, at subsequent increases in duration of the rest periods, the difference remained practically at the same level.

At dronedarone treatment, positive dynamics of the inotropic response of the papillary muscles was obtained on rest periods (Figure 3). So, after rest of 4 s, postrest contraction was 138 ± 1.92% that was by 11% higher than in control conditions. Statistically significant difference (p < 0.05), as with amiodarone, was obtained at rest period of 6 s. However, at longer rest duration, increase of inotropic response of the muscles treated with dronedarone was less than one with amiodarone. Reliable (p < 0.05) difference between amiodarone and dronedarone effect was obtained for rest period duration of 20 s and more. So at rest period duration of 60 s difference between effects of the drugs was 17% (p < 0.05).

### 3.5. The Effect of Amiodarone and Dronedarone on Decay of Potentiation Induced by Rest

**Figure 4:**
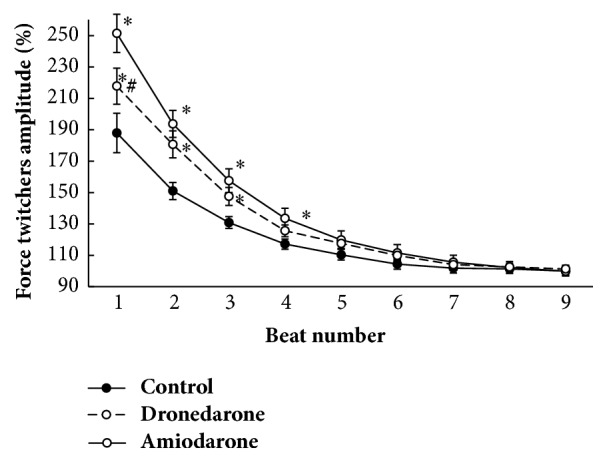
Influence of drugs on the dynamics of potentiation fall after rest period of 60 s. *∗*p < 0.05 versus control. #  p < 0.05 versus amiodarone.

Dynamics of recovery of papillary muscle contractions after 60 s rest is presented in Figure 4. The amplitude of potentiated contraction after rest was reduced with each subsequent contraction. The amplitude of contraction of muscle strips after amiodarone treatment differed from control values up to the first 4 cycles inclusive and after treatment with dronedarone, up to the first 3 contractions (p < 0.01). However, in the case of intact muscles and in the case of muscles treated with amiodarone or dronedarone, decrease in inotropic response to initial values occurred to the 9th regular contraction-relaxation cycle.

To estimate recirculating fraction of calcium ions, the decay coefficient of contraction force, potentiated with rest period of 60 s, was calculated [29] (Figure 4). For this, linear regression of the amplitude of the n-th contraction versus amplitude of the n + 1 contraction was built [28, 29]. Graph showing calculation of the coefficient of potentiation decay (CPD) of inotropic response after 60 s rest did not present. Slope angle of the obtained graph was the coefficient of potentiation decay (CPD) of contraction amplitude after rest period of 60 s. Under control conditions, CPD was 62.9 ± 2.85%, at amiodarone treatment was 64.1 ± 3.31%, and at dronedarone was 67 ± 2.71%; significant differences between the groups were not revealed, although on the background of dronedarone, the checkpoint in comparison with the control had tendency to increase in value.

## 4. Discussion

The results of our studies have shown that amiodarone and dronedarone do not equally affect the parameters of single contraction-relaxation cycle. Amiodarone decreased contraction amplitude (Tmax), as well as velocity parameters of contraction-relaxation cycle, having no effect on cycle duration, time of developed tension, and relaxation time. Dronedarone, as well as amiodarone, decreased contraction amplitude and velocity parameters of contraction and relaxation; at the same time it increased contraction-relaxation cycle period. These data indicated that dronedarone increased the refractory period, based on drug properties. Decrease of contraction amplitude of rat myocardium after amiodarone and dronedarone treatment that was observed is in agreement with the weak negative inotropic effect of class III antiarrhythmic drugs [30, 31]. A similar effect can be result of free Ca^2+^ decrease in cardiomyocyte myoplasm during contraction as a result of the inhibitory effect of these drugs on slow calcium channels of cell membrane and/or their adrenoblocking action [5, 32]. In addition, we found significant differences at comparison of effects of dronedarone and amiodarone on extrasystolic contractions. The dronedarone suppressed extrasystolic contractions to a greater degree than amiodarone did, which can testify more significant decrease of excitability of cardiomyocyte membranes. The same effect confirms the fact that after dronedarone treatment extraordinary impulse after 0.2 s did not cause the extrasystolic contraction. The fact of influence of amiodarone and dronedarone on the effective refractory period does not raise doubts; however, in our study amiodarone did not significantly alter this index. Probably, the used dosage was insufficient for manifestation of this effect, in contrast to dronedarone. Most likely, this is connected with the fact that dronedarone has 10-fold stronger blocking effect on sodium currents [INa] than amiodarone [33].

The results of this study reveal that amiodarone and dronedarone are capable of amplifying postextrasystolic and postrest potentiation of inotropic response of the papillary muscles. According to modern concepts, both postextrasystolic and postrest potentiation of the inotropic response characterize functional activity of the sarcoplasmic reticulum [14, 27]. In this regard, it can be assumed that amiodarone and dronedarone are able to act not only on the receptors and ion channels of the sarcolemma, but also to modulate the state of the intracellular Ca^2+^ transporting systems of cardiomyocytes. This circumstance, possibly, is important factor determining the high antiarrhythmic activity of these drugs. It is known that the intracellular mechanisms of trigger activity that induce the arrhythmias are connected with overload of cardiomyocytes with calcium ions [19, 34]. Trigger activity occurs as a result of early and delayed afterdepolarization (EAD and DAD, respectively). Early afterdepolarizations occur during repolarization phase plateau at increase of action potential (AP) duration as a result of the reactivation of Na^+^ and/or Ca^2+^ channels of the L type [35, 36]. As it is known, amiodarone and dronedarone blocked influx Na^+^ and Ca^2+^, channels of L type [6, 31], that probably determines their ability to arrest the trigger activity of early afterdepolarizations.

The mechanisms of the formation of trigger activity of DAD are also of great importance. DPDs appear after repolarization phase of AP. It has been established that DADs are induced by spontaneous diastolic leakage of calcium ions from the SR [18, 37]. It is proved that the amount of Ca^2+^ released from the SR correlates with DAD amplitude [37]. Spontaneous diastolic leakage of Ca^2+^ from the SR can occur as a result of overload of the intracellular depot with calcium in the intact myocardium [37] or as a result of alteration of functions and/or structure of the calcium channels (ryanodine receptors) of cardiomyocytes at heart failure [15, 38, 39]. It is known that the hyperactivation of sympathetic nervous system can lead to hyperphosphorylation of protein kinase of ryanodine receptors and subsequent reorganization of their macromolecular structure [40, 41]. As a result, calcium channels of SR partially transfer in open state that contributes to current of Ca^2+^ leakage from SR during diastole [21, 41, 42]. Such calcium “waves” can induce membrane depolarization and, accordingly, trigger activity. Prevention of trigger activity of such mechanism by known antiarrhythmic drugs is very difficult. However, it is possible to reduce the Ca^2+^ influx into cell using calcium channel blockers. At the same time, calcium “waves” can trigger themselves by mechanism of Ca^2+^-activated release of Ca^2+^ from the SR [15, 43]. In this case, use of calcium channel blockers cannot influence the intracellular change of calcium level. Thus, despite the fact that both drugs possess a weak negative inotropic effect, they increased postextrasystolic and postrest contractions. It is known that an increase in contraction amplitude is the result of an increase in calcium ion amount involved in contraction process [14]. Probably, these drugs are capable of activating Ca^2+^-ATPase, promoting reuptake of Ca^2+^, and/or increasing the speed of Ca^2+^ transport from the uptake sites to the release sites in the SR. On the other hand, these drugs can also reduce the leakage of Ca^2+^ currents, allowing for the retention of calcium ions in the SR during rest periods and augmenting potentiation of postrest contractions. These effects can contribute to decreasing the amount of excess calcium ions in the sarcoplasm and to decreasing the arrhythmogenic Ca^2+^ oscillations in the cell, thereby providing antiarrhythmic actions in DPD-induced arrhythmias.

As it is known, the coefficient of potentiation delay reflects the recirculation of intracellular fraction of calcium ions [28, 29]. Results of study have shown that an antiarrhythmic drug of the III class—amiodarone and dronedarone—does not practically influence recirculating fraction of intracellular Ca^2+^, calculated as the coefficient of the force contraction decay potentiated by 60 s rest.

The results of our study have indirectly shown that the amiodarone and dronedarone are capable of exerting influence on SR functional activity and thereby exerting influence on the intracellular transport of Ca^2+^. This assumption corresponds to our previous studies using caffeine [44]. Caffeine stimulates the release of Ca^2+^ from the SR, causing a short-term increase in contraction amplitude, followed by the suppression of contractions amplitude due to SR Ca^2+^ pool depletion [45, 46]. We showed that preliminary treatment of muscles with caffeine caused the cancellation of postextrasystolic and rest period-induced potentiation [44]. Additionally, the increase in postextrasystolic and postrest potentiation induced by amiodarone was completely abolished by caffeine. These data indicate that the amiodarone-induced increase in postextrasystolic and rest period-induced potentiation is due to an increase in SR Ca^2+^- accumulation.

The results of the present study indicate that the force-interval dependence in muscles treated with amiodarone and dronedarone has similar dynamics. Furthermore, postextrasystolic and postrest potentiation was increased by these drugs. These data may reveal an additional property of the III class antiarrhythmic drugs, dronedarone and amiodarone, to modulate the intracellular transport of Ca^2+^ by influencing the functional activity of the SR. This property is undoubtedly positive and may promote the high antiarrhythmic efficiency of these drugs.

Thus, dronedarone and amiodarone are able to increase postextrasystolic and postrest potentiation; however dronedarone reduces the excitability of the sarcolemma of cardiomyocytes to a greater extent than amiodarone, but effect of amiodarone on postextrasystolic and postrest potentiation is more pronounced than one of dronedarone.

Our results allow considering an amiodarone as more effective antiarrhythmic drug at arrhythmias genesis delayed afterdepolarization-induced trigger activity, and dronedarone is more effective drug for preventing an early afterdepolarization.

## Figures and Tables

**Table 1:** Influence of amiodarone and dronedarone on the contraction-relaxation cycle parameters.

	n	Тmax, %	+dТ/dt, %	-dT/dt, %	t, %	t1, %	t2, %
Before	14	100	100	100	100	100	100
Amiodarone	14	90 ± 3,3*∗*	92 ± 2,8*∗*	87,1 ± 3,55*∗*	102 ± 2,3	102 ± 2,69	104 ± 2,80
Before	8	100	100	100	100	100	100
Dronedarone	8	92 ± 2,90*∗*	89 ± 2,69*∗*	81 ± 3,01*∗*	114 ± 4,69*∗*#	106 ± 2,80	116 ± 3,38*∗*#

The values of contraction-relaxation cycle parameters after amiodarone or dronedarone treatment were estimated as percentage of the baseline of contraction-relaxation cycle parameters. *∗* P < 0.05 versus baseline. # P < 0.05 versus amiodarone.
